# “Just fighting for my life to stay alive”: a qualitative investigation of barriers and facilitators to community re-entry among people with opioid use disorder and incarceration histories

**DOI:** 10.1186/s13722-023-00377-y

**Published:** 2023-03-21

**Authors:** Kim A. Hoffman, Emma Thompson, Marina Gaeta Gazzola, Lindsay M. S. Oberleitner, Anthony Eller, Lynn M. Madden, Ruthanne Marcus, David E. Oberleitner, Mark Beitel, Declan T. Barry

**Affiliations:** 1 Department of Medicine, Oregon Health and Science University, Portland, OR USA; 2 APT Foundation, New Haven, CT USA; 3 Rutgers New Jersey Medical School, Rutgers, Newark, NJ USA; 4 Department of Emergency Medicine, New York University School of Medicine, New York, NY USA; 5 Oakland University William Beaumont School of Medicine, Rochester, MI USA; 6 Department of Internal Medicine, Yale School of Medicine, New Haven, CT USA; 7 Department of Psychology, University of Bridgeport, Bridgeport, CT USA; 8 Department of Psychiatry, Yale School of Medicine, New Haven, CT USA; 9 Child Study Center, Yale School of Medicine, New Haven, CT USA

**Keywords:** Methadone, Opioid use disorder, Incarceration, Re-entry, Qualitative

## Abstract

**Background:**

During the period of community re-entry immediately following release from jail or prison, individuals with opioid use disorder (OUD) face structural barriers to successful re-entry and high risk of overdose. Few published studies investigate experiences in the immediate period (i.e., first 24 h) of re-entry among people with OUD.

**Aim:**

To understand the barriers and facilitators to treatment and reintegration of people with OUD during the initial transition from carceral settings back into the community.

**Methods:**

From January–December 2017, we conducted 42 semi-structured qualitative interviews with patients with a history of incarceration who were receiving methadone at a not-for-profit, low-barrier opioid treatment program. Interviews probed participants’ community re-entry experiences immediately following incarceration. Interviews were transcribed and analyzed using a Thematic Analysis approach.

**Results:**

The main themes described the experiences during the 24 h following release, reacclimating and navigating re-entry barriers, and re-entry preparedness and planning. Participants noted the initial 24 h to be a period of risk for returning to substance use or an opportunity to engage with OUD treatment as well as a tenuous period where many lacked basic resources such as shelter or money. When discussing the subsequent re-entry period, participants noted social challenges and persistent barriers to stable housing and employment. Participants overall described feeling unprepared for release and suggested improvements including formal transition programs, improved education, and support to combat the risk of overdose and return to substance use after incarceration.

**Conclusions:**

In this study that qualitatively examines the experiences of people with incarceration histories and OUD enrolled in methadone treatment, we found that participants faced many barriers to community re-entry, particularly surrounding basic resources and treatment engagement. Participants reported feeling unprepared for release but made concrete suggestions for interventions that might improve the barriers they encountered. Future work should examine the incorporation of these perspectives of people with lived experience into the development of transition programs or re-entry classes.

## Introduction

Incarceration increases risk of return to substance use and creates significant barriers to receiving evidence-based treatment for opioid use disorder (OUD) [1–3]. Upon release from a carceral setting, there is a substantial elevation in risk of return to use and overdose [4]. Risk for substance-related death is high in the initial weeks following release from incarceration and substance overdose is the leading cause of death after release, making the first 24 h post release critical for vulnerable individuals [2, 5–7]. The initial weeks after release from jail or prison, termed “re-entry,” are associated with economic uncertainty, fractured social and family relationships, and housing instability [8–11]. Previous qualitative research documents the challenges formerly incarcerated individuals experience post-release [12–16]. For example, O’Brien et al., describes post-release challenges related to meeting basic survival needs such as housing and employment, as well as reconnecting to children and other family members [17]. For people with OUD who are incarcerated, there is added complexity related to need for treatment and recovery; re-entry provides an important window to connect to evidence-based treatment.

Rigorous research demonstrates that medications for opioid use disorder [MOUD] with an opioid receptor agonist (methadone), partial agonist (buprenorphine), or opioid antagonist (naltrexone) can facilitate recovery from OUD [18, 19]. These life-saving pharmacotherapies, often delivered at opioid treatment programs (OTPs) along with other treatment and recovery services, can facilitate reintegration and reduce risk of return to substance use and overdose. However, there is limited research on barriers and facilitators that individuals with OUD experience immediately following release from incarceration and how these experiences directly or indirectly affect access to OTPs.

Return to substance use during the re-entry period is influenced by social environment, financial situation, housing setting, emotional support, and comorbid medical conditions [12, 20]. In addition to lack of support and limited resources immediately post-incarceration, individual attitudes towards MOUD can reduce the likelihood of evidence-based treatment engagement and increase risk of nonmedical opioid use and death [20]. However, existing structural support for pre-release planning is limited and rarely accounts for barriers or personal priorities [11]. Understanding the barriers and facilitators of transitioning into society after incarceration for people with OUD may assist in improving the existing resources and infrastructure for this population. Given the nascent state of the research regarding experiences of re-entry among persons with OUD, a qualitative approach that solicits first-hand accounts of personal experiences may be particularly beneficial [21]. The aims of this study were to investigate the re-entry planning experience, perceived levels of preparation to return to life after incarceration, and the challenges faced during the re-entry period among people in an OTP with a history of incarceration.

## Methods

### Context

The study was conducted at the APT Foundation, Inc. (APT), a not-for-profit community-based organization affiliated with Yale School of Medicine. APT offers primary and psychiatric care along with substance use disorder (SUD) treatment, including four licensed OTPs. APT utilizes the “open access model,” a low-barrier treatment approach which aims to eliminate common OUD treatment barriers, such as long wait times, requiring abstinence from other substances, or being able to pay for treatment [22].

### Sampling strategy

Participants were recruited voluntarily using flyers posted at all APT clinic sites, as well as clinician referral. Inclusion criteria were: (1) English fluency; (2) current OTP enrollment; (3) incarceration history defined as spending one day minimum in jail or prison in one’s life. One hundred individuals contacted the team for screening. Prospective participants were placed on a contact waitlist in the order in which they contacted the study in response to flyers. A research assistant sequentially contacted people on the waitlist to schedule an interview until the interviewers determined that data saturation had been met; fifty-one were contacted for interview scheduling; forty-five participated in interviews, which were concluded due to thematic saturation. Three interviews were excluded from the final sample due to technical difficulties.

### Ethical issues pertaining to human subjects

The study was reviewed and exempted by the Yale University IRB and was approved by the APT Foundation Board of Directors. Participants provided informed consent and were compensated with a $25 gift card. Data and interviews were deidentified by trained research assistants not involved with data analysis. Demographic survey data was not connected to participant interviews or medical records.

### Data collection and processing

Interviews were conducted privately as a single appointment at the participant’s treatment site by experienced qualitative interviewers: a licensed clinical psychologist (DTB) and a graduate student in public health (AE). The semi-structured interview guide included the following domains: (1) key problems faced after release; (2) description of the first 24 h post- release; and (3) experiences with housing and employment post release. The interviews ranged from 25 to 55 min (mean 38 min). Interviews were audiotaped, and research assistants transcribed the transcripts verbatim, removing identifying information. Participants completed anonymous demographics surveys.

### Data analysis

Qualitative analyses focused on interview questions about participants’ experiences during community re-entry (Appendix 1). Data were analyzed using Thematic Analysis [23] with a mixed deductive and inductive approach. The study team developed a list of deductive top-level codes after reading transcripts, with codes grouped into the following overarching themes: (1) experiences during the initial 24 h after release from carceral settings; (2) reacclimating into society: housing, employment, and relationships; and (3) re-entry challenges. Contents of each coding category were reviewed to ensure agreement on the nature of respondents’ responses to the interview. The first author (KH) led the qualitative analysis; she has extensive experience implementing qualitative investigations. Study team members KH, MG, and ET further refined the coding framework to include inductive secondary themes within each overarching theme. Using ATLAS.ti Mac (Version 22.1.0) and ATLAS.ti Web (Version 3.19.1.-2022-06-20), two coders (KH and ET) coded the data with this framework [24]. An inter-coder agreement process was conducted in which 20% of the data were double-coded and agreement was 88%. Results are reported following Standards for Reporting Qualitative Research [25].

## Results

### Demographics

Participants were 42 (19 female, 23 male) patients enrolled in OTPs who ranged in age from 26 to 70 years, with a mean (SD) age of 42.9 (10) years. Twenty-four percent (n = 10) self-identified as Black, 19% (n = 8) as Hispanic, and 57% (n = 23) as White. Participants’ self-reported number of times in jail or prison varied from 1 to 60 (median 4.0, 25th IQR = 2.0, 75th IQR = 8.3). The range of self-reported number of family members (parents, siblings, children, cousins, uncles, or aunts) who had ever been in jail or prison was 0 to 26 (median 4.0, 25th IQR = 1.0, 75th IQR = 5.0).

### Theme 1: participant experiences during the initial 24 h after release

Participants described the difficulties they encountered during the initial hours after release related to returning to opioid use, prioritizing their health and safety, and MOUD treatment-seeking.

### Subtheme 1: seeking substances or treatment

Participants reported a range of activities during their first 24 h post-incarceration. Commonly, they described a dilemma around a return to substance use. A contrast emerged between respondents who immediately sought substances and those who sought treatment or recovery group support:

First day after release… freedom! [laughing] But also… you have to make a decision whether you’re gonna use or not. When people say ‘oh I’ve been three months clean’ or something like that and they were in jail… yeah well, you’re clean because the drugs weren’t available to you… you gotta make that decision whether you’re gonna start all this shit again. And I chose this time, I chose to use… so it was like none of that jail time never even happened.

-Participant 9

Participants juxtaposed the draw and the dangers of an immediate return to substance use. Some struck a “balance” in their approach:

It’s good to get out, you wanna celebrate… but you don’t wanna party too hard I guess because if you go back to jail it’s just gonna be worse than last time. Every time you go, it’s a little longer usually because your record builds up.

-Participant 18

I did a little partying but I wasn’t gonna run wild and run back to what I used to do. I was gonna do better.

-Participant 22

Participants described using various substances, most commonly cigarettes, alcohol, and cannabis, within the first 24 h after release:

Even though I was clean for a while… the first thing I wanted to do was get an Oxycontin…basically the first thing I did.

*-*Participant 3

Conversely, approximately a third of respondents viewed their release as a catalyst for “running” to MOUD treatment—usually methadone—or recovery settings. One respondent even walked the considerable distance from the courthouse to the OTP.

I got released, the minute I came running to the program, tell them what happened, they gave me my dose, I still had a bottle in my house, and whoop, I was happy.

-Participant 2

### Subtheme 2: freedom versus abandonment

Participants’ responses differed based on whether or not they received structured support during release, such as through family, MOUD treatment, halfway houses, or social programs. Without such supports, MOUD treatment-seeking was not a priority; rather, survival needs were utmost in their minds. Participants who were not released to a structured environment overwhelmingly reported worry about a lack of support and were unable to focus on treatment-seeking. They had few or no resources, while simultaneously were burdened with many immediate concerns.

My first day when I got released, they dropped me off in front of the courthouse at 5:30 in the morning and handed me my stuff and said there ya go… You don’t get money to get released. Family has to meet you at the bus stop…if I didn’t have money, I wasn’t getting home… there’s no help…. they say they help you, but they don’t. They just leave you to fend for yourself.

-Participant 6

This lack of resources or assistance often necessitated sleeping outside immediately upon release:

I guess it [sleeping outside] was okay… I’m not uncomfortable outside. I feel like I can breathe when I’m out there. So, it wasn’t bad for me. The first night was good for me I guess I would say, I got things accomplished here and at the housing, so my first night was good…I felt satisfied with what I had to do.

-Participant 4

Interviewer: Okay and then that resulted in you staying where?

Respondent: On a park bench.

Interviewer: Even though it may sound like a strange question what was it like?

Respondent: Horrible.

-Participant 6

Conversely, those who could return to a home described feelings of excitement and freedom. They framed the home environments positively due to immediate basic needs being met:

Came home, ate, hung out, didn’t have anyone telling me when I could piss, shit, eat, shower, whatever…It was great. Loved every minute of it.

-Participant 30

Another respondent described enjoying the newfound freedom, but that this prompted the need to find structure:

It was exciting…to come home. But I was also scared because once you’re there [incarcerated], you get used to that schedule so when you come home, you gotta get used to everybody else’s schedule cause now you’re incorporating yourself into their life again.

-Participant 38

In contrast to those released to a less structured environment, those who were mandated to a particular setting such as residential treatment or a halfway house reported stability, but with undesirable features such as isolation and feeling “closed off” [Participant 14]. One respondent noted that they were unable to visit family or help with family errands. Others highlighted more extreme feelings of restriction, as though they were still in a carceral environment as exemplified in this respondent’s experience with MOUD residential treatment,

I hate to say it this way, I truly do, but I went from being locked up in one place to being locked up in another place because I was mandated to be there…so I hate to put it that way because I was thankful I was able to go there to get out of jail, but it was basically being from locked up one place to being locked up in another pace. But at least when I got released that day I knew exactly when I was gonna be on the street. When I was gonna be back home. I knew exactly when- when you’re in jail you don’t know. You have no idea.

-Participant 10

### Subtheme 3: social, logistic, and material supports

Participants recounted facing significant logistical and resource-related challenges during the initial 24 h after release, which complicated access to treatment. Common issues included transportation, shelter, and money. While some experienced high levels of family and social support, most discussed the inability to access basic necessities.

I don’t even know where I stayed that night. I didn’t have anything, like no clothes or nothing at that time so I think I might have stayed at a friend’s house.

-Participant 1

Most participants experiencing high support found this through family, though in some cases social programs filled this gap and prevented return to substance use and jail:

They got me right at the YWCA and the next thing we went to motor vehicles to get my IDs—if it wasn’t for that program, I’d have been… probably right back in jail within a week. But they helped me. They took me shopping for clothes… they got me the basic needs… They got me a bus pass. They signed me up for my doctors. I went to see my doctor so I could get my medications.

-Participant 11

More often, logistical and emotional support came from family members. The reconnection brought relief along with complex emotional reactions:

Oh excitement, I went to the park. I went to every family member that I could… My mom came and got me. That was awkward because I had no help but she did let me stay at her house so… we’re okay…—I had to rebuild her trust.

-Participant 24

The day of my release my father picked me up from jail. That was tough, we had a long conversation in the car. Uh he was crying, I was crying.

Participant 23

I remember everyone came. My family picked me up. I got to see my daughter right away. It was nice… it felt like a homecoming.

Participant 28

Most participants mentioned characteristics of their sleep when asked about their first night after release. All remarked on this as a salient experience with roughly half remarking on the high quality of their sleep:

I slept though. I slept like a baby cause those beds up there you cannot sleep on.

*-Participant 19* Other participants noted difficulty adjusting:

The excitement, anxiety just got to me so bad that, I don’t know, I couldn’t sleep. If I slept two hours that was a lot. I still couldn’t sleep that whole next day. It took me over a week or better to get adjusted. Once that week went by, my body kinda came down to reality. Freedom [laughing], freedom.

-Participant 15.

### Theme 2. reacclimating: experiences with relationships, housing, and employment

#### Subtheme 1: return to pre-incarceration behaviors and substance use

Concerns of *“*picking up with bad relationships*”* [Participant 25] were often cited as a challenge to seeking MOUD treatment and recovery; familiar people and places were described as triggers for return to substance use:

Cause people come out and they think ‘oh you know I’m clean, I’ve been in jail 30 days, or 40 days, I’m clean you know like it’s just gonna be that simple’. It’s not that simple, when you go back out and shit is around you, and if you’re around the same people again you know it like they all the same- people, places, and things. Cause if nothing changes, nothing changes.

-Participant 4

Similarly, respondents reported that resuming past patterns or contending with past trauma caused re-acclimation challenges and return to pre-incarceration behaviors and substance use:

My main challenges, one it’s my own thinking. My own. Drugs and alcohol, just life itself. I have a lot of things that I haven’t addressed. People have died, in my life, just here alone.

-Participant 31

While respondents were eager to return to society after their release, they described mental and behavioral barriers:

Acclimating back into society, being around normal people. You often act a certain way for so long… you have to speak an entirely different language in prison. And sometimes people carry that out when they get out and it takes them a while to learn how to actually socialize normal again.

-Participant 16

Most respondents reported motivation to shift their mindsets and habits to avoid returning to substance use:

I just learned some things when I got out of prison and I’m still learning everyday…Fighting addiction…Trying to find steady employment…Fighting…just fighting for my life to stay alive.

-Participant 29

Just trying to get my life on. I’m getting old, and it’s time to change. I just want a better life that’s all.

-Participant 34

Part of the described difficulty in re-entry was rebuilding family relationships: respondents frequently cited “family trust” [Participant 24] as one of their main challenges and motivators for trying to succeed on a new path and working on their recovery from substance use:

Getting my family to trust me was the hardest thing…Trust me again and forgive me.

-Participant 12

#### Subtheme 2: persistent barriers to stable housing and employment

After being released, some participants returned to stable employment and housing environments but most encountered structural and logistical barriers. Those who had stable housing or employment prior to incarceration found that even brief periods in jail created substantial interruptions:

Struggling to get a job, struggling to get housing or an apartment. Maybe struggling with homelessness. It’s just not easy to come out of jail. Even just going in for a week, it’s not easy to come out and just continue, especially if you had a job and you went to jail and you don’t have that job anymore. You know you need to find a job and hope that your record doesn’t affect that.

-Participant 18

Stigma related to background checks were universal barriers to housing and employment. One respondent described background checks as a “death sentence” [Participant 16]. Others said it was their “biggest challenge” since release, preventing both employment and, in turn, their ability to pay rent.

Background checks have kept me from getting good jobs… When they say if you’re honest it won’t exclude you from employment, but then when you’re honest it does exclude you. So, it’s a catch 22. I can’t win. It’s hard because they say when you’re on parole you have to get a job or you’re going back to prison, but places don’t wanna hire felons.

-Participant 17

For many participants, housing was a major challenge. Many adopted varied strategies to avoid homelessness including halfway houses, sober living or inpatient residential programs, shelters, staying with friends and family, going “couch to couch, hotel to hotel” [Participant 16].

Well, I be staying with my brothers, my cousin, back and forth.

-Participant 34

I’ve lived with my grandmother, for a few months and I have lived with boyfriend, where I am now. And I had a stint where I was homeless.

-Participant 31

While some viewed living in a shelter as a “step up” from living “on the streets” [Participant 39], others described how shelters were restrictive and too much “like jail” [Participant 30]:

When you’re at a shelter you have to bring all your belongings with you every day, no matter where you go, what you do. And you have to be out of the building from 7–5 and you try to go for a job or going to talk to somebody, whatever you have to bring all your things with you, I mean that’s ridiculous, suitcases and bags. Come on—really?

-Participant 1

Other barriers included lack of training and education, particularly for participants who were incarcerated for significant periods of time during which technology advanced:

It’s been very difficult [to find work]. Not only is my criminal record an issue, I have no education. I quit in 10th grade so… I fill out applications everyday online. At least online I fill out a few every day, if not going places and filling them out, but no one ever calls me back. … it’s been very hard.

-Participant 13

I got two years of college in human services but seems everything changed. You gotta know computers all that stuff, I don’t know computers, it’s hard for me to get a job, now that I’m old.

-Participant 37

### Theme 3: preparedness and planning

#### Subtheme 1: preparedness related to return to substance use and MOUD treatment

When asked how participants could have been better prepared to leave the carceral setting, the universal response was better coordination with logistical, social, and medical supports, especially substance treatment, “because without the treatment I believe that there’s a lot of people that are gonna be dying,” [Participant 5].

They could have people like counselors or something and help you prepare, just to help you find a job so you don’t return to the same thing you were doing. If you just get released and you got nothing to do, you’re just gonna do the same thing.

-Participant 3

Start process of like housing and everything beforehand they actually have done things now, like they help you get identification and stuff before you leave now, I just didn’t have enough time to do it cause I was released from the court. But if I knew I was gonna be being released, they woulda had ID for me and everything. I would say that before releases, they need to talk to people about housing. And programs like set it up before you even leave, have an appointment for you… If people had an appointment to come here [methadone clinic] from county, might not get high. If they came right here and did what they’re supposed to do to get on the program.

-Participant 4

One participant noted that an easy preventative measure would be to provide individuals leaving prison or jail with a list of local mutual support meetings or even a sponsor. Another, who had been on methadone while incarcerated remarked:

I wish maybe they informed me a little bit about you know what to do, where to go. Cause I was on methadone, like where to go and how to proceed with that.

-Participant 23

In rare cases, facilitation with substance treatment occurred with excellent effect:

My case manager from [substance treatment program] was visiting me, coming in for legal visits and I also kept in touch because [names] from the [program] are at the jail every day. I was doing [program] groups while I was there in the prison so I was able to keep in contact with [names] from here and they helped me to facilitate my release as far as what I need to do from day one, going to [treatment location] and you know the other things I would need to do as far as staying in contact with [program] and getting medicated as soon as I was released which kept me from relapsing.

-Participant 25

#### Subtheme 2: set up for success

When asked what they wished they had known prior to re-entry, a majority of respondents had a simple request: to be told of their release date with sufficient notice to prepare logistically and emotionally for re-entry. Many participants felt that “They just sprung it on me*”* [Participant 19], and felt more notice would have helped them feel ready:

I wasn’t prepared because they just called me in the middle of the morning and said you gotta go …I wish I was told ahead of time I was leaving… If I would have known, I would have been prepared to know where I was going and how everything works.

-Participant 5

“Many respondents indicated that there is an unmet need for transitional programs that navigate release logistics, especially related to connecting to health and treatment services:

If your release date is coming up, I think they should give you that application for Husky [Connecticut Medicaid] or whatever cause you can automatically be approved for that for 6 months cause you’re being released from prison, obviously you don’t have a pot to piss in, you know. So, I think that’s something they should do in jail so when you come home, you’re insured, you’re capable of going out and getting all the things you need. Going to the doctor, going to the clinic, whatever you need instead of having to go home, and then wait however long it takes to do it in the real world you know. I don’t think it could hurt to start the process sooner.

-Participant 14

Another related sentiment was feeling abandonment due to the manner of release, which resembled *“moving cattle”* [Participant 16]. Echoing the challenges highlighted in previous sections, respondents indicated they were released “*with no help and no resources”* [Participant 21], yet face higher scrutiny by community supervision or police and risk reincarceration:

You’re kind of sent out with no resources, no money, nowhere to go, and expected on the conditions of your probation to have a job and have somewhere to live and have a phone number and be able to have transportation to parole and probation. And have all these conditions that you absolutely have to abide by in order not to go back to jail and no way to buy any of these things for yourself. Basically, it’s an impossible situation. And that’s why, you know like 85% of people end up back.

-Participant 21

Another critical element to successful re-entry is engagement in outpatient MOUD treatment. One facilitator of post-incarceration treatment engagement was receiving treatment while incarcerated:

*I was doing APT groups while I was there, we had group at Whalley Avenue every other Friday for the people that were on the APT methadone clinic in the prison… I was able to keep in contact with [names] from here and they helped me to facilitate my release as far as what I need to do from day one, going to [OTP clinic location]…the other things I would need to do as far as staying in contact with APT and getting medicated as soon as I was released which kept me from relapsing.*

-Participant 2

Some of the participants identified other hurdles to engaging in MOUD treatment post-release. For example, a small number of participants expressed reticence towards MOUD modalities, despite continued engagement. One participant noted “I don’t wanna be on methadone the rest of my life” [Participant 22] while another opined that methadone treatment could just be a “revolving door” [Participant 16]. Other participants who desired continuity of MOUD treatment could not access it due to lack of facilities in the area where they lived.

*New Haven’s a good place that I like. Coming out of New Britain it was like no help for a recovering addict. There’s no places, no methadone clinic. You didn’t know about none of that. It’s very isolated.*

-Participant 42

## Discussion

This was one of the first qualitative studies to examine perspectives on community re-entry, including the first 24 h following release from incarceration, among individuals engaged in outpatient methadone treatment. There were several important findings. First, we found that people with OUD viewed the re-entry period as a time of possibility and risk regarding substance use and treatment, during which many struggled to meet basic needs. Second, both in the initial post-release period and overall re-entry episode, respondents reported challenges surrounding housing, employment, and social relationships. Third, participants reported feeling unprepared for release and suggested changes to improve the re-entry process for people with OUD, including improved treatment facilitation. Our findings build on the few prior studies examining the incarceration experience among people with OUD by evaluating the first 24-h post-release and re-entry preparation and focusing on individuals who are no longer incarcerated and engaged in methadone treatment [14, 20, 26].

We found that the initial 24 h after release were crucial to participants’ decision to use substances or seek treatment. Our participants suggest that people enrolled in methadone treatment facing incarceration may be amenable to more structured linkage to treatment during this period. This finding is supported by other qualitative studies [27]. For example, in a qualitative investigation of the Fresh Start Re-entry Program in Connecticut, formerly incarcerated individuals described how their case managers prepared them for release through services designed to facilitate their return to the community [28]. Qualitative research with carceral staff support these findings. For example, in interviews with jail staff, one study’s respondents stressed the importance of facilitating MOUD post release [29]. Likewise, in interviews with medical and administrative staff at MOUD programs serving jail-referred individuals, respondents noted the need for more structured care coordination for jail-to-community-based MOUD treatment [30].

Though we are unaware of other studies which emphasize the first 24 h after release, it is generally known that re-entry is a vulnerable period for return to substance use and overdose [12, 31, 32]. Respondents reinforced this finding and noted that in addition to struggling with decisions about resuming substance use, they also encountered numerous logistic challenges including navigating transportation and shelter, consistent with other studies about re-entry for people with SUD [20, 33]. These barriers provide an opportunity for actionable policy and practice. For example, Kaplowitz et al. found that participants suggested transportation options such as subsidized bus passes could facilitate treatment in the community post-release [26].

Other studies demonstrate a range of material barriers after release for individuals with and without OUD, including a lack of both personal resources and information on how to access resources [26, 34–36]. It is notable that many participants felt that the lack of resources ultimately set them up to return to prison or jail, emphasizing how social and structural factors influence criminal legal system involvement [37]. An element of Welsh and Rajah’s (2014) qualitative framework “re-entry work” is echoed in respondents’ stories—the difficulty of caring for oneself, establishing housing, and renewing family relationships [38]. In another qualitative study, people experiencing homelessness were afraid they would return to substance use after release [26]. Moreover, Hyde et al., found that veterans recently released from prison expressed “transitional anxiety” which accompanied seeking housing and employment [39]. Participants noted that background checks for employment and educational programs that exclude persons with felony convictions limit options for people with a criminal record. Loosening these restrictions would offer more opportunities for employment and education for these individuals.

Respondents also reported behavioral, emotional, and relationship-related difficulties reacclimating after release. They reported experiencing fear of returning to past social and behavioral patterns and of being released into settings that make substance use impossible to avoid. In a similar study, Kaplowitz et al. noted that such variables can impact engagement and retention in MOUD [26]. Additionally, participants perceived inadequate notice of their release, an absence of transitional programs to prepare them for re-entry, and inadequate education about how to avoid return to substance use post-incarceration. Engagement with discharge planners prior to release could provide information about services available upon re-entry into the community and information on the risk of overdose if substance use is reinitiated. Availability to meet upon release with peer navigators or people with prior incarceration experience to guide them through the process of re-entry and to assist with OTPs may alleviate some of these difficulties with reacclimating. Future research should examine how implementing such interventions prior to or upon release might impact re-entry outcomes. While prior research demonstrates that re-entry can be an emotionally tumultuous and high-risk time for individuals with OUD, more work is needed to identify the best program structures to implement to improve re-entry outcomes [40–42].

Our findings extend and support the existing literature on the lack of transitional support and the paucity of referrals to MOUD or mental health counseling during the release period. They also bolster documentation of a need for both services, by highlighting suggestions made by people with lived experience of incarceration and OUD who were able to engage in MOUD treatment despite facing multiple barriers to obtain MOUD treatment [43–47]. Consistent with prior study findings, participants noted that transition programs helped individuals with OUD to re-enter society by providing material support, a network of relationships, and continuity of MOUD care [27, 41]. The re-entry period is a time with high risk of overdose death, and better access to MOUD during this time is critical in reducing mortality [2, 6, 48, 49]. Lack of treatment due to breaks in MOUD continuity may lead to substance use and in turn an increase in mortality risk [46, 50, 51]. Salem et al. advocate for the development of multi-level interventions at the individual, program, and societal level help bridge the gap during re-entry [52]. Transition programs, linkage to MOUD, and re-entry interventions such as case management services are associated with decreased recidivism and retention in treatment within this population [53, 54]. In their systematic review of findings from qualitative evaluations of community re-entry programs, Kendall et al. found case workers were key to program success; suggesting that policy and practice to strengthen their role could lead to improved outcomes [27].

Access to MOUD by incarcerated persons varies widely by location and carceral setting [55]. On April 5, 2022, the US Department of Justice issued new guidance clarifying that across-the-board policies banning MOUD violate the Americans with Disabilities Act [56]. The release of this guidance accelerated an ongoing shift at the state level towards providing incarcerated individuals access to MOUD. For example, the US Attorney's Office in Massachusetts announced on April 1, 2022 that it had coordinated with state and local officials to ensure that all available types of MOUD would be provided in all state and county correctional facilities [57]. However, not all states have acted in response to this guidance, and many incarcerated persons still face difficulty obtaining MOUD; some facilities only provide access to Naltrexone or utilize MOUD solely to manage withdrawal, while others restrict MOUD access to only certain populations of prisoners [58]. Nonetheless, policymakers can utilize existing resources to comply with this guidance, such as adoption of model laws, and thereby quickly and meaningfully expand MOUD access [59]. Access can also be improved through updating federal methadone regulations, elimination of the Medicaid payment exclusion for incarcerated individuals, and through creating alliances with community-based treatment [58]. Improving MOUD access while incarcerated remains critical, with preliminary analysis of Connecticut's pilot program indicating that participation statistically reduced post-release non-fatal overdose and increased individual's likelihood of continuing methadone treatment after release [60].

Carceral settings are an often overlooked component of the opioid epidemic and present policy and practice opportunities for assessing individual risk for return to use, overdose education, and MOUD enrollment. Increasingly, research shows that inclusion of carceral settings in the continuum of care, such as pre-release initiation of buprenorphine, can overcome logistical barriers such as those described by study respondents [61, 62]. Transitional initiatives such as those examined in the Justice Community Opioid Innovation Network (JCOIN) can facilitate discharge planning, connect individuals to vital services post-release, and promote recovery from SUD [63]. Other helpful policy changes can be found in the Post-Release Opioid-Related Overdose Risk Model which include providing naloxone training and take-home kits to those leaving a criminal justice setting with a risk of return to opioid use; coordination between criminal justice settings, healthcare, and community-based treatment; and community partnerships to assist with housing and job placement [64]. Other possibly helpful initiatives include Transitions Clinic Network (TCN) that is designed to meet needs through a national network of medical homes for those recently released from incarceration with chronic health problems [41].

This study has limitations. Participants were treatment-engaged individuals so generalizability to other formerly incarcerated populations with OUD may be limited. While the coded responses from the interviews yielded potentially important themes related to participants’ experiences, we did not collect data from others to corroborate these themes. Participants’ experiences were not bifurcated on whether they had received their sentence or were unsentenced during their incarceration; there are differences in experiences with SUDs and re-entry concerns between these populations that this study did not address. Additionally, we did not systematically collect quantitative data on the duration, dates, and setting (e.g., prison versus jail) of each individual’s incarceration. If experiences between the two settings differ, this study did not capture them. It is possible that individual’s recall of their experiences could be affected by different time lengths, and this is a limitation of our work. To protect anonymity, participants’ answers were not linked to demographic or clinical characteristics; these sources of information may have provided an enriched context for interpreting the study.

## Conclusions

In the first hours after release from incarceration, people with OUD faced many barriers to community re-entry, including problems accessing methadone treatment engagement, shelter and employment, and difficulty managing social relationships. Future investigations should consider examining the feasibility of widespread transitional program implementation with a focus on education about the overdose crisis within carceral settings, supporting and maintaining relationships during and post-incarceration as well as linkage to outpatient MOUD programs as interventions to reduce mortality during re-entry and enable people with OUD to return safely to the community (65–67).

## Data Availability

Upon request.

## Qualitative interview questions

- 15. When were you released?
- 19. What are key problems that people face after they have been released from jail or prison?
- 20. What have been your main challenges since being released? “key problems that you and others face after release” collapsed 19 and 20.
- 21. Describe the first day after your release.
- 22. Describe the first night after your release **collapse 21/22 into “First 24 h post-release”.**
- 23. Since being released, where have you lived? [If unclear, “Since being released, did you have permanent housing?”].
- 24. Since being released, where have you worked? How many jobs? [If not fulltime work, “Have you wanted full-time work?” “How easy or difficult has it been to find work?”] **chunk 23 and 24 “experiences with housing and employment post-release”.**
- 25. How prepared were you for your release?
- 26. What do you wish that you had known or been told before you were released?
- 27. How could jails or prisons better prepare people for life after release?

## Appendix 2. Background/Demographics Form

Please answer all the questions below.

- 1. How old are you? _____.
- 2. Are you male ___ or female ___?
- 3. What is your ethnic or racial background? _________________.
- 4. How many members of your family (parents, siblings, children, cousins, uncles or aunts) have been in jail or prison? _________.
- 5. How many members of your family (parents, siblings, children, cousins, uncles or aunts) ever had problems with alcohol or drugs? _________.
- 6. How many members of your family (parents, siblings, children, cousins, uncles or aunts) ever had problems with heroin or painkillers? _________.
- 7. How many close friends do you have? _________.
- 8. How many of your close friends have served time in jail or prison? _________.
- 9. How many of your close friends ever had problems with alcohol or drugs? _________.
- 10. How many times have you been in jail or prison? _________.
